# Herpes Zoster Infection of Orofacial Region in a Geriatric Patient: A Case Report

**DOI:** 10.7759/cureus.62049

**Published:** 2024-06-10

**Authors:** Shivangi Navghare, Swapnil Mohod, Sourabh B Shinde, Vaidehi Thakre

**Affiliations:** 1 Oral Medicine and Radiology, Sharad Pawar Dental College and Hospital, Datta Meghe Institute of Higher Education and Research, Wardha, IND

**Keywords:** infection, lesion, trigeminal nerve, varicella-zoster virus, herpes zoster

## Abstract

The varicella-zoster virus reactivates to cause the “herpes zoster” (HZ). ‘‘Varicella-zoster virus’’ (VZV) termed as ‘‘HHV-3’’ or ‘’human herpesvirus-3’’ infection causes herpes zoster. Varicella, the primary form of the virus, is chickenpox, and the secondary form of the virus is herpes zoster also called shingles. During prior chicken pox episodes, this virus enters the body through cutaneous nerve endings and becomes dormant in the dorsal root ganglia. It sometimes affects the orofacial region and appears as unilaterally distributed burning pain, multiple, painful vesicular lesions, and ulcerations. Immunocompromised people are more likely to have disseminated zoster, which is defined as the involvement of three or more dermatomes. These are most likely to occur in elderly, immunocompromised patients, patients undergoing cancer chemotherapy, patients on immunosuppressants, and patients suffering from AIDS. This is a study of a male geriatric patient, aged 74 years, who reported unilateral pain, swelling, as well as multiple ulcerations on the left side of his face, extraorally as well as intraorally. The case was diagnosed as a herpes zoster infection involving V1 and V2 dermatome of the trigeminal nerve.

## Introduction

The DNA virus called “varicella-zoster virus” (VZV) can cause both primary as well as recurrent infections. It affects people with any age group but generally affects the elderly. Neuropathic pain, headaches, malaise, and disturbed sleep are examples of prodromal symptoms. In addition to neuropathic pain in the involved dermatome, a localized, itchy, vesicular rash caused by herpes zoster (HZ) usually appears unilaterally along a few nearby sensory nerves [1]. The degree of immunosuppression and age is related to a rise in the prevalence of herpes zoster infection (HZI). Those with lower levels of cell-mediated immunity are more likely to have HZI. This encompasses the elderly, individuals undergoing chemotherapy or steroids, patients with HIV, and patients with lymphoma [2]. Three stages are evident in clinical terms as follows: pre-eruptive, acute exudative, and chronic. Pre-eruptive symptoms include headaches, photophobia, and malaise in addition to burning pain with affected dermatomes for two to three days before cutaneous eruptions [3]. Shingles is a painful, usually unilateral rash eruption [2]. During the acute exudative phase, several uncomfortable, umbilicated vesicles form.

These blisters often burst, become ulcerated, and eventually dry up. This stage is the most contagious. Frequently, nonsteroidal pain relievers do not relieve severe pain. Acute eruptions can recur for three to four weeks. Pain may last longer [3]. In the affected dermatome, the lesions are initially concentrated at a few locations. The vesicles frequently combine into larger, fluid-filled lesions as the disease worsens [1]. In the chronic phase, severe pain lasts more than a month. Patients may develop paraesthesia and shock-like sensations. This pain will last from several days to months and is disabling. For confirmation when HZ-type pain without rash is suspected, polymerase chain reaction testing can be useful [3]. The first trigeminal nerve division is typically impacted, resulting in herpes zoster ophthalmicus. When the trigeminal nerve's first division is affected, lesions develop on the scalp, forehead, and upper eyelid. With the involvement of second-division lesions on the mid-face and upper lip and lesions on the lower lip and lower face with the involvement of third-division trigeminal nerve.

## Case presentation

A 74-year-old elderly male reported to the outpatient department with a complaint of swelling on the left side of his face for three days. The swelling was initially smaller and progressed to its current size. The patient also experienced pain for two days. The pain was dull aching and intermittent which gets aggravated on chewing as well as stimulated with hot and cold stimuli. The pain relieves on its own. The patient also gives a history of mucous discharge from the outer canthus of the left eye for one day. The patient was referred to the department of oral medicine for further treatment. The patient has been hypertensive for the past 50 years and is on medications for the same. The patient also has bronchial asthma for the past 10 years and is on medications for the same. The patient did not provide details of medications. The patient visited a private dental clinic for pain and swelling where medications were prescribed that could not relieve him of the symptoms. The patient gave a history of extraction in the upper right and left back region of the jaw one year back at a private dental clinic which was uneventful. The patient had a habit of kharra (a combination of tobacco, betel nut, and lime) chewing five to six times a day for the past 15-16 years. The patient is not allergic to any drug known to him till now.

**Figure 1:**
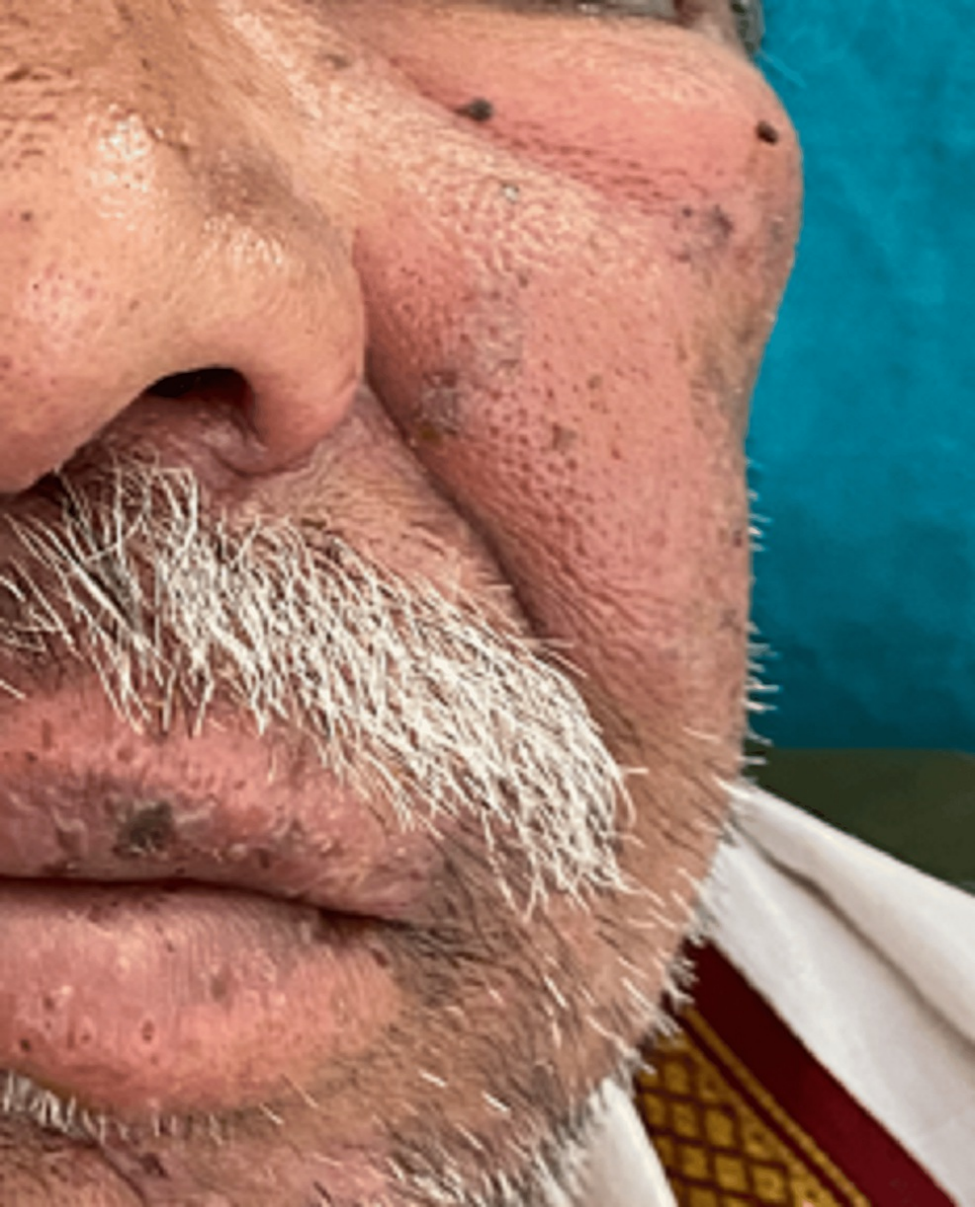
Extension of swelling on the left side of face from left lower eyelid to lower lip.

On extraoral examination, asymmetry of the face was observed due to swelling across the left side of the face that extends superoinferiorly from the left lower eyelid to the lower lip and mediolaterally from the lateral wall of the nose to the left zygoma region (Figure 1). The swelling was roughly oval of size 5x4 cm approximately with irregular margins, having a smooth surface and dense crusting seen on the upper labial mucosa. It was reddish in color. A pattern of unilateral, clustered blisters, ulcers, and scabs was seen on the left half of the face. Dense crusting over the upper lip was seen (Figure 1).

**Figure 2:**
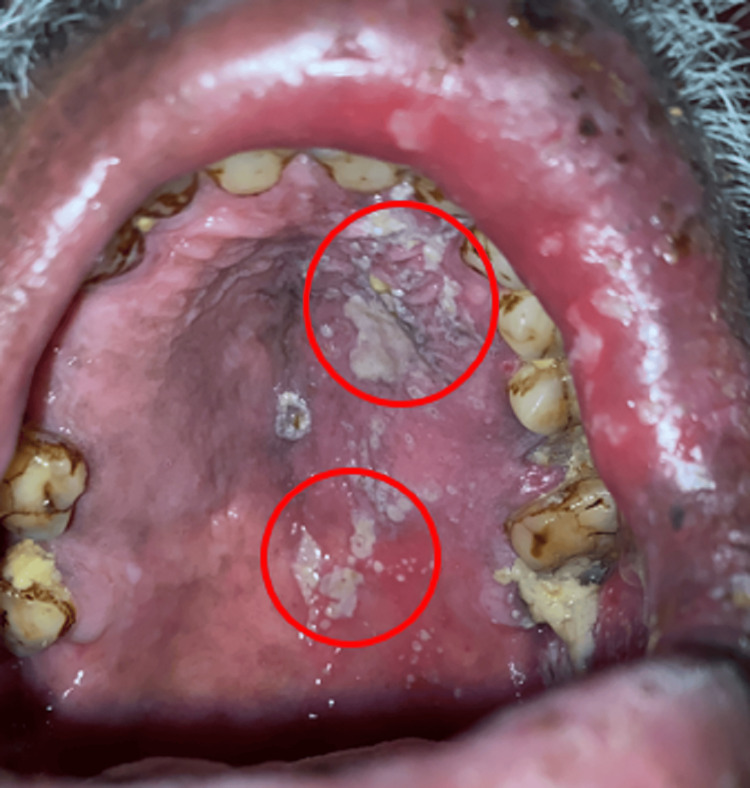
The marked area shows a unilateral diffused whitish patch and ulcers on the left side of palate and crusting over upper lip.

On palpation, the local temperature was raised and tenderness was present. It had a soft consistency with a smooth surface texture. Temporomandibular joint movements were bilaterally smooth and synchronous. No tenderness was present on palpating temporomandibular joint and its associated muscles. A single, firm, mobile, and tender lymph node in the left submandibular area, measuring roughly 0.5x0.5 cm, was palpable. On intraoral examination, coalescing ulcers were seen on the left buccal mucosa of 35-36 region, approximately 3x2 cm in size, having a smooth surface with central sloughing with erythematous margins suggestive of aphthous-like ulcer. A diffused whitish patch was seen on the attached gingiva of 45-46 region suggestive of leukoplakia which might be due to the habit of betel quid keeping in the left buccal vestibule region. Unilateral diffused whitish patches and ulcers were also seen on the left side of the palate (Figure 2). The white coating is seen on the dorsal surface of the tongue. Crusting over upper lip is also seen (Figure 2). On clinical examination and unilateral distribution of the lesion, a provisional diagnosis of "herpes zoster infection" was given. The patient was advised glycated hemoglobin (HbA1c) test but patient failed to comply and was not tested due to monetary reasons.

**Figure 3:**
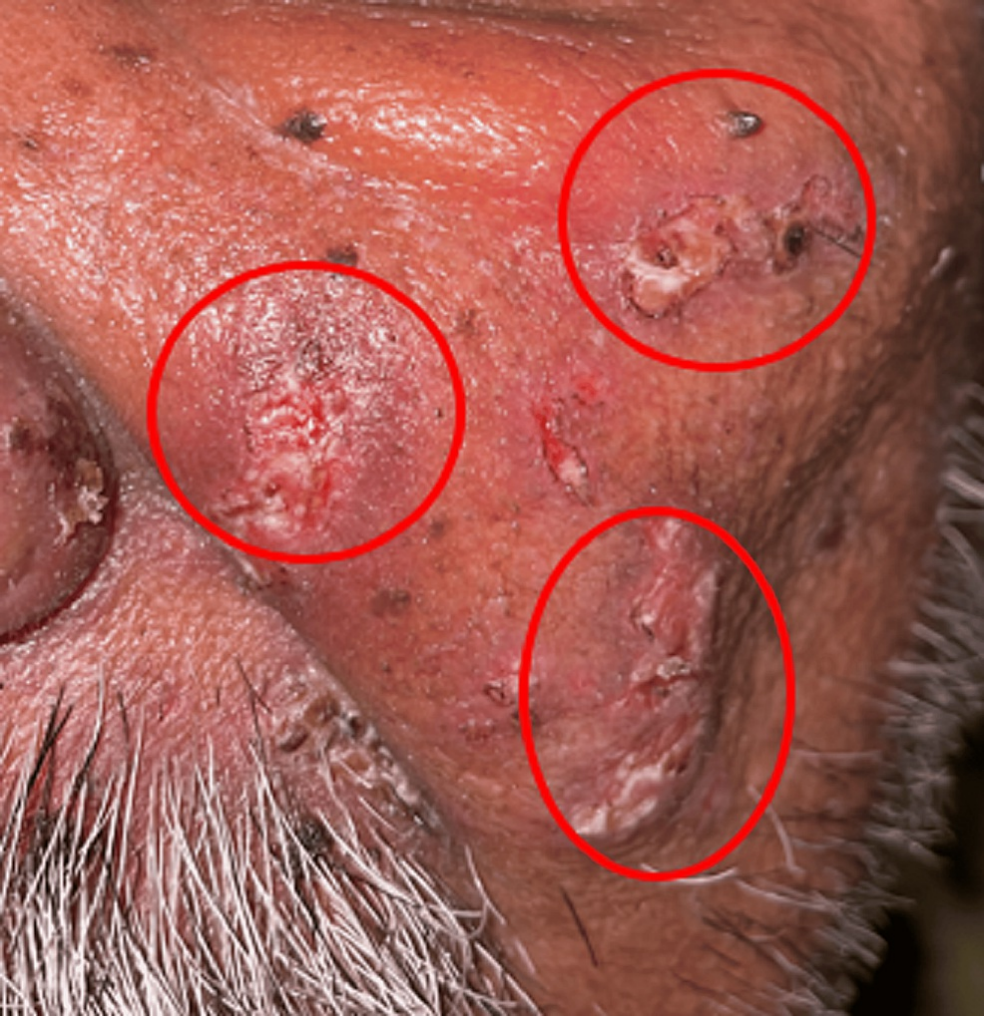
The marked area shows reduced swelling and healing of the lesion on the left side of the face extraorally on the first follow-up.

**Figure 4:**
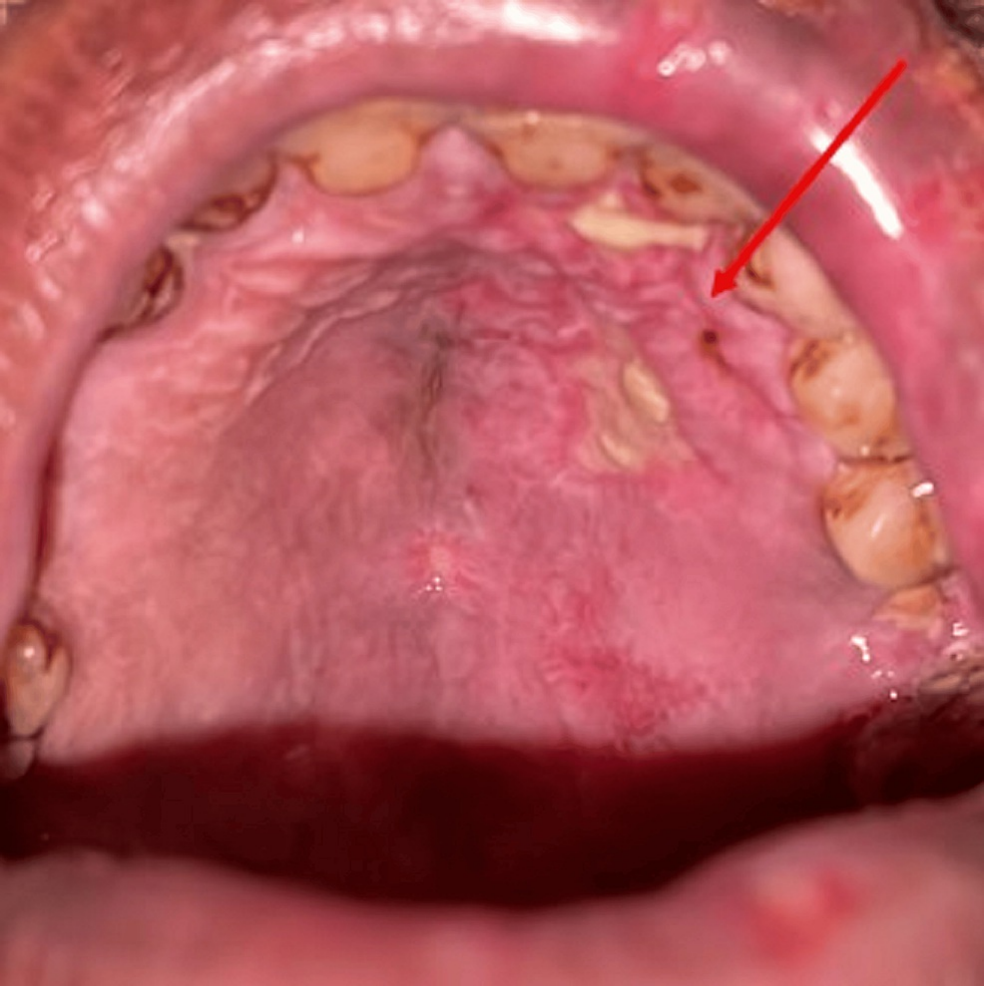
Arrow showing healing of lesion on the left side of palate intraorally on first follow-up.

**Figure 5:**
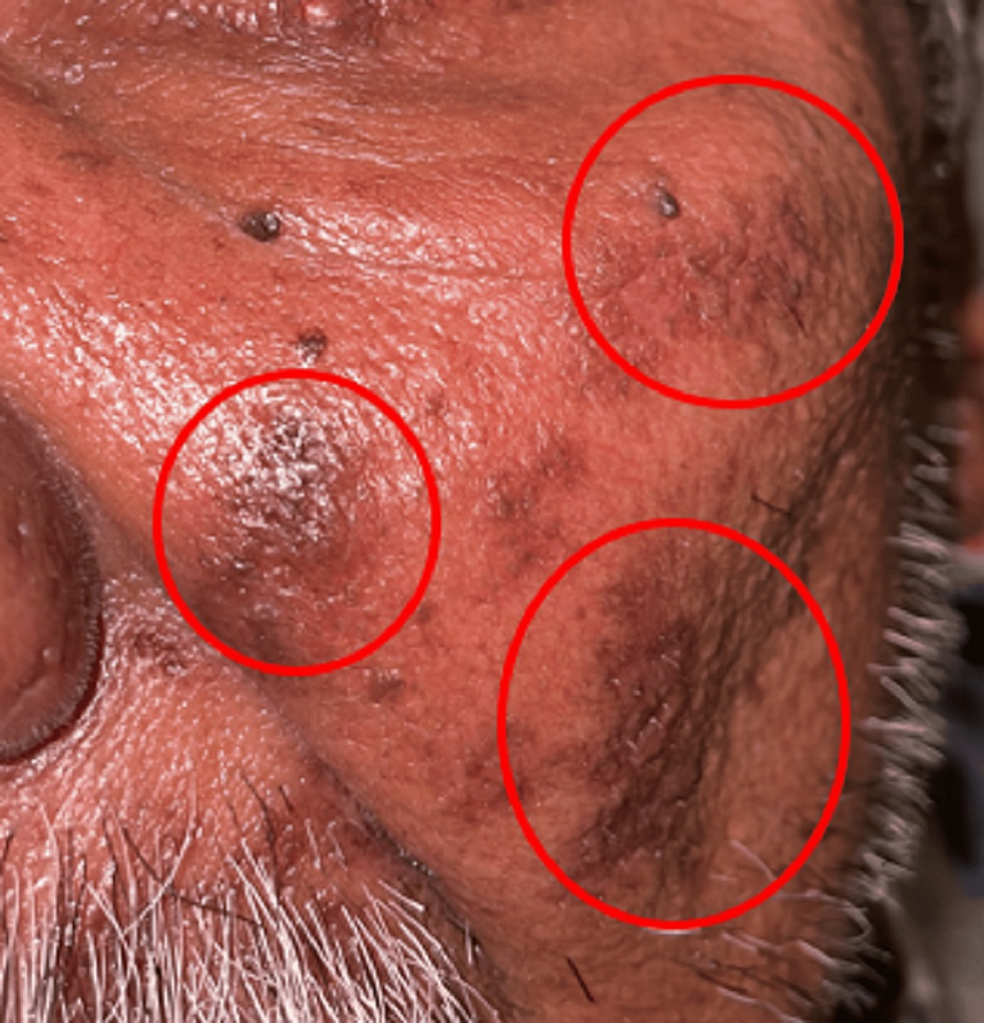
The marked areas show healing of lesions with formation of dry scabs extraorally on the second follow-up.

**Figure 6:**
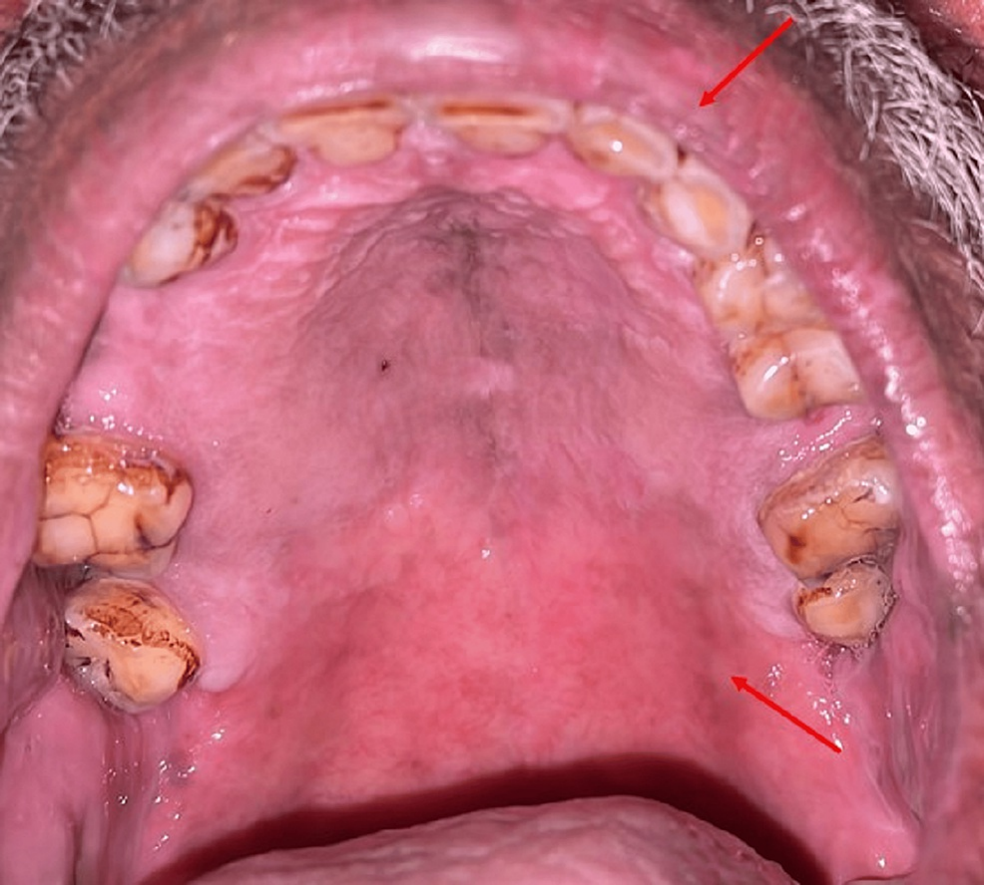
Arrow showing complete healing of lesion on the left side of palate and reduced crusting on upper lip.

The patient was instructed to consume a bland diet and periodic hydration, tab. valacyclovir 1000 mg three times daily for five days, tab. Neurobion Forte (multivitamin supplement) twice a day for five days, topical application of triamcinolone acetonide four times a day for five days, and topical application of acyclovir ointment thrice daily for five days were also prescribed. A follow-up of five days was given. On the first follow-up, reduced facial swelling and healing of the lesion on left side of face extra orally was seen (Figure 3). Reduced discharge from the eye and reduced ulceration on the lip and oral mucosa was seen and pain subsided to some extent. Healing of the lesion on the left side of palate was seen intraorally (Figure 4). A 20% reduction in the intraoral as well as extraoral lesions was noted. Valacyclovir was continued for the next three days. On the second follow-up, extraoral lesions as well as intraoral lesions disappeared leaving a dry scab (Figure 5). Complete healing of the lesion seen on the left side of the palate and reduced crusting on the upper lip was seen (Figure 6). Valacyclovir was discontinued. The patient was advised regular follow-up.

## Discussion

The human alpha herpesvirus causes both "chicken pox" as well as shingles. Chickenpox also known as varicella is a common childhood infection characterized by fever, viremia, and sporadic vesicular skin lesions [4]. Herpes zoster is not caused by exposure to the varicella-zoster virus. On the other hand, those with varicella zoster can spread the virus to contacts who are seronegative. Not herpes zoster, but varicella, develops in these contacts [5]. The most common ways for the virus to spread are through direct contact with lesions or through the air [6]. Herpes zoster usually manifests as a mild to moderate burning and tingling under the skin of a specific dermatome, which occasionally goes along with headache, malaise, chills, nausea as well as fever. In some instances, numbness may also be present. The severity of shingles pain is so severe, causes excruciating muscle spasms to be triggered by the slightest breeze or touch [7]. Three to five days after they first appear, the lesions typically start to dry and scab. The disease usually takes seven to 10 days in total, but it can take a few weeks for the skin to heal normally. Vesicular lesions occur in the mouth when the V2 and V3 branches of the "trigeminal nerve" are involved [8]. Differential diagnoses of oral HZI include herpetiform recurrent aphthous stomatitis as well as primary or secondary infection with herpes simplex virus (HSV).

The most common complication of shingles is post-herpetic neuralgia (PHN) which causes intense and burning pain in nerves as well as in skin. The majority of patients with post-herpetic neuralgia experience nerve pain or allodynia along with intense, ongoing, or sporadic burning or lancinating pain [5]. Inability to perform daily activities, depressive disorder, losing weight, sleep disturbances, and persistent tiredness are all possible consequences of severe post-herpetic neuralgia. Pain might be felt beyond the affected dermatome [9]. Secondary bacterial infections caused by *Staphylococcus aureus* and *S. pyogenes*, which include septicemia, zoster gangrenosum, cellulitis, and necrotizing fasciitis, are the general complications that arise after post-herpetic neuralgia. Antibiotic-resistant patients and the elderly are more vulnerable to bacterial infections. Cellulitis may result in scarring and necrosis. The potentially fatal condition of necrotizing fasciitis can be made worse by streptococcal toxic shock-like syndrome [5].

The healthcare provider should provide HZ with the necessary education and support in addition to treatment. Adherence to therapy and patients' health is enhanced by giving a thorough description of the condition, which includes the possibility of transmission of the virus to those who did not have an earlier infection of chickenpox [10]. The first line of treatment for treating herpes zoster as an antiviral agent is acyclovir. In patients with herpes zoster infection antivirals like acyclovir, famciclovir, or valacyclovir shorten the time of viral shedding and the formation of new lesions while hastening the healing of the rash. For all immunocompetent HZ patients who meet any of the following requirements, systemic antiviral therapy is highly advised as the initial course of treatment: ages 50 years or older, severe rash, severe pain, or non-truncal involvement are the four qualifying conditions. In addition, oral corticosteroids also have a beneficial effect on acute pain [11]. For many years, patients with HZ have been treated for severe pain with sympathetic and epidural nerve blocks. In the present case, steroids were used topically; systemic application was contraindicated as the patient is a known case of hypertension and bronchial asthma. Additionally, the extent of the lesion and severity of the condition pointed towards a systemic condition that the patient might have, for example, diabetes mellitus, which exaggerated the lesion beyond its normal appearance.

## Conclusions

As discussed, the occurrence of HZI is suppressed by the immune system so it generally occurs in patients with low immunity. Diagnosing the complications of HZI is a bit challenging among oral physicians due to their varied appearance. Management of the patient with antivirals within 70-72 hours after the onset of the rash results in a reduction in post-herpetic complications. Maintaining immunity and stress and getting the varicella vaccine can prevent the occurrence of shingles. Thorough knowledge of the infection will help in early diagnosis and prevention of serious complications of the disease. Follow-up should be given to the patient to avoid further complications.
